# Prevalência e Características Relacionadas de Pacientes com Eletrocardiograma com Padrão de Brugada em Santa Catarina, Brasil

**DOI:** 10.36660/abc.20190542

**Published:** 2021-08-09

**Authors:** Mariana Sarmento Militz, Andrei de Souza Inacio, Harley Miguel Wagner, Aldo von Wangenheim, Alexander Romeno Janner Dal Forno, Daniel Medeiros Moreira

**Affiliations:** 1 Universidade do Sul de Santa Catarina PalhoçaSC Brasil Universidade do Sul de Santa Catarina, Palhoça, SC - Brasil.; 2 Universidade Federal de Santa Catarina FlorianópolisSC Brasil Universidade Federal de Santa Catarina, Florianópolis, SC - Brasil.; 3 Instituto de Cardiologia de Santa Catarina São JoséSC Brasil Instituto de Cardiologia de Santa Catarina, São José, SC - Brasil.; 4 SOS Cardio FlorianópolisSC Brasil SOS Cardio, Florianópolis, SC - Brasil.

**Keywords:** Sindrome de Brugada, Eletrocardiografia/métodos, Obesidade, Prevalência, Arritmias, Hereditariedade, Epidemiologia

## Abstract

**Fundamento:**

A síndrome de Brugada é um distúrbio arritmogênico hereditário caracterizado pela presença de características eletrocardiográficas específicas com ou sem sintomas. Os pacientes apresentam risco aumentado de morte súbita por fibrilação ventricular. A prevalência desse padrão eletrocardiográfico difere de acordo com a região estudada. Porém, informações epidemiológicas, incluindo a população brasileira, são escassas.

**Objetivo:**

Avaliar a prevalência do padrão eletrocardiográfico da síndrome de Brugada e o perfil epidemiológico associado a ela.

**Métodos:**

Estudo transversal que incluiu 846.533 registros ECG de 716.973 pacientes do banco de dados de eletrocardiograma (ECG) da Rede de Telemedicina de Santa Catarina por um período de quatro anos. Todos os exames foram ECG de 12 derivações convencionais (sem V1 e V2 em posições altas). Os exames identificados com o diagnóstico de “Síndrome de Brugada” (tipos 1 e 2) foram revisados por um eletrofisiologista. Foram considerados significativos valores de p<0,05.

**Resultados:**

Apresentavam padrão potencialmente consistente com ECG do tipo Brugada 83 pacientes. Destes, 33 foram confirmados com padrão de Brugada tipo 1, e 22 com tipo 2, após reavaliação. A prevalência de ECG do tipo 1 de Brugada foi de 4,6 por 100.000 pacientes. O ECG do tipo Brugada 1 foi associado ao sexo masculino (81,8% vs. 41,5%, p<0,001) e menor prevalência de obesidade (9,1% vs. 26,4%, p=0,028).

**Conclusões:**

Este estudo mostrou baixa prevalência de ECG do tipo Brugada no sul do Brasil. A presença de ECG com padrão Brugada tipo 1 esteve associada ao sexo masculino e menor prevalência de obesidade que a população geral.

## Introdução

A síndrome de Brugada (SB) é uma doença arritmogênica hereditária caracterizada pela presença de características eletrocardiográficas específicas com ou sem sintomas clínicos. Os pacientes são, em sua maioria, jovens, e apresentam risco aumentado de morte súbita por fibrilação ventricular (FV).^1^^,^^2^ Essa entidade clínica foi descrita pela primeira vez em 1992, quando os irmãos Brugada relataram 8 casos de pacientes com FV idiopática que haviam tido morte cardíaca súbita abortada. Esses pacientes apresentavam ECGs com supradesnivelamento do segmento ST nas derivações precordiais direitas na ausência de cardiopatia estrutural, distúrbio eletrolítico ou isquemia.^3^

A SB pertence a um grupo de canalopatias causadas por mutações que ocorrem em genes que codificam ou regulam os canais de sódio do músculo cardíac.^4^ Este padrão de transmissão genética tem uma característica autossômica dominante com mutações dos genes SCN5A e SCN10A ligados ao fenótipo de Brugada.^4^^,^^5^

O diagnóstico da SB pode ser feito por meio de ECG de 12 derivações, demonstrando elevação do ponto J nas derivações precordiais direitas.^1^^,^^2^ No entanto, a verdadeira prevalência da síndrome entre a população em geral é complicada de estimar porque alguns pacientes têm padrão eletrocardiográfico de Brugada transitório.^2^ Acredita-se que a SB seja responsável por 2 a 12% de todas as mortes súbitas e pelo menos 20% dos óbitos de pacientes com corações estruturalmente normais.^1^^,^^2^ Estudos realizados em países asiáticos têm demonstrado maior prevalência de padrão eletrocardiográfico de Brugada em comparação com outras regiões.^6^^,^^7^ Por outro lado, são escassas as informações epidemiológicas que incluam a população brasileira. Este estudo foi realizado para identificar a prevalência e as características relacionadas de pacientes com padrão eletrocardiográfico de Brugada em Santa Catarina-Brasil.

## Métodos

Neste estudo, foram incluídos 846.533 registros eletrocardiográficos de 716.973 pacientes do banco de dados de eletrocardiogramas (ECG) da Rede Catarinense de Telemedicina, uma ferramenta eletrônica que ajuda os profissionais de saúde a visualizarem e diagnosticarem exames de imagem remotamente. A rede está conectada a unidades básicas de saúde e regiões remotas de mais de 250 cidades do Estado de Santa Catarina, Brasil. Estima-se que o sistema processe mais de 17.000 ECGs por mês.^8^

Foram considerados elegíveis todos os pacientes que realizaram o ECG de doze derivações e tiveram seus prontuários processados na Rede Catarinense de Telemedicina no período de agosto de 2010 a dezembro de 2015.

A maioria dos exames foi realizada em regime ambulatorial, principalmente em unidades básicas de saúde com indicação de avaliação cardiovascular na atenção primária (dados não apresentados). Todos os exames do nosso banco de dados foram avaliados por um cardiologista treinado e todos aqueles identificados com o descritor “Síndrome de Brugada” como diagnóstico foram revisados por um segundo cardiologista especializado em eletrofisiologia.

Utilizou-se o Relatório de Consenso dos Critérios Eletrocardiográficos Atuais para o Diagnóstico do Padrão de Brugada como base para a identificação do padrão eletrocardiográfico.^9^ Os ECGs foram reavaliados pelos seguintes critérios: Tipo 1 (padrão arqueado) com supradesnivelamento de ST inicial ≥2 mm descendo lentamente e côncavo ou retilíneo em relação à linha de base isoelétrica, com onda T simétrica negativa em V1-V2, e Tipo 2 (padrão sela de cavalo) — origem alta (r') ≥2 mm em relação à linha isoelétrica e seguido por supradesnivelamento do segmento ST convexo em relação à linha de base isoelétrica com elevação ≥0,05 mV com onda T positiva/plana em V2 e variável de onda T em V1.

Considerou-se apenas o primeiro exame confirmado de cada paciente. Para comparação, utilizamos o primeiro teste de todos os outros pacientes que realizaram ECG no mesmo período e não foram diagnosticados com SB. Por se tratar de um estudo retrospectivo, sem acesso direto aos dados clínicos dos pacientes, objetivou-se estimar a prevalência do padrão eletrocardiográfico da síndrome de Brugada. No entanto, não é possível determinar a real prevalência da síndrome, uma vez que não podem ser descartados exames que eram fenocópias da síndrome de Brugada, por exemplo.^10^ Também comparamos dados epidemiológicos entre os grupos de exames como idade, sexo e prevalência de doenças prévias, incluindo diabetes, dislipidemia, hipertensão, doença renal crônica, doença arterial coronariana, doença de Chagas e infarto agudo do miocárdio prévio.

### Análise Estatística

As análises estatísticas foram realizadas com o software SPSS 13.0 (SPSS Inc., Chicago, IL, EUA). Utilizou-se o teste de Kolmogorov-Smirnov para avaliar a normalidade das variáveis contínuas: todas apresentaram distribuição normal e, a seguir, foram apresentadas como média e desvio padrão. As variáveis categóricas foram apresentadas por números absolutos e porcentagens. As variáveis quantitativas entre os grupos estudados foram avaliadas pelo teste *t* de Student não pareado. Utilizou-se o teste exato de Fisher para testar a associação entre proporções. O nível de significância adotado foi *p*<0,05.

## Resultados

O presente estudo incluiu 846.533 exames de 716.973 pacientes. Entre eles, 83 pacientes apresentaram um padrão possivelmente compatível com padrão eletrocardiográfico de Brugada. Excluímos 129.560 exames por pertencerem aos mesmos pacientes. Após reavaliação dos exames com diagnóstico de SB por um especialista em eletrofisiologia, foi possível confirmar 55 ECGs com padrão de Brugada. Destes, 33 exames foram diagnosticados como padrão de Brugada tipo 1 e 22 exames como padrão de Brugada tipo 2. O grupo de comparação (sem diagnóstico de SB) tinha 716.918 pacientes.

A prevalência do padrão eletrocardiográfico de Brugada tipo 1 ou 2 foi de 7,6 por 100.000 pacientes. A prevalência do padrão eletrocardiográfico de Brugada tipo 1 e padrão de Brugada tipo 2 foi de 4,6 e 3,0 por 100.000 pacientes, respectivamente.

As características da amostra são apresentadas na Tabela 1. A média de idade dos pacientes no grupo de Brugada tipo 1 ou 2 foi de 48,0±16,0 anos. No grupo Brugada tipo 1 ou 2, 78,2% dos participantes eram do sexo masculino, e no grupo Brugada tipo 1, 81,1% dos participantes eram do sexo masculino, apresentando proporção significativamente maior do que a população geral avaliada (41,5%) com p<0,001 para fins de comparação.

**Tabela 1:** Características demográficas e clínicas da população estudada

Variáveis		Padrão de Brugada tipos 1 e 2 n (%) média±DP	Padrão de Brugada tipo 1 n (%) média±DP	Ausência de padrão de Brugada n (%) média±DP	Valor de p*	Valor de p†
Sexo						
	Masculino	43 (78,2)	27 (81,8)	297131 (41,5)	<0,001	<0,001
	Feminino	12 (21,8)	6 (18,2)	419603 (58,5)	–	–
Idade (anos)		48.0±16,0	48.0±15,5	50.0±19,6	0,431	0,568
Altura (cm)		168.0±11,0	166.8±12,6	163.3±11,1	0,002	0,067
Peso (Kg)		72.6±15,6	72.7±17,2	73.6±17,4	0,664	0,776
IMC (kg/cm2)		25.4±4,2	25.7±4,5	27.5±5,6	0,001	0,084
HAS		19 (34,5)	12 (36,4)	253469 (35,4)	0,994	1,000
Obesidade		4 (7,3)	3 (9,1)	188961 (26,4)	0,001	0,028
DM		6 (10,9)	3 (9,1)	54732 (7,6)	0,312	0,738
Tabagismo		1 (1,8)	1 (3,0)	51645 (7,2)	0,185	0,730
IAM		0 (0,0)	0 (0,0)	6960 (1,0)	>0,999	>0,999
Dislipidemia		2 (3,6)	1 (3,0)	63229 (8,8)	0,234	0,361
DAC		6 (11,0)	3 (9,1)	92999 (13,0)	0,841	0,794
DC		0 (0,0)	0 (0,0)	359 (0,1)	>0,999	>0,999
DRC		0 (0,0)	0 (0,0)	2819 (4,0)	>0,999	>0,999
DPOC		0 (0,0)	0 (0,0)	8837 (1,2)	>0,999	>0,999
Revasc		1 (1,8)	1 (3,0)	4962 (6,0)	0,268	0,171

* *Brugada tipos 1 e 2 vs. Ausência de Brugada,*

† *Brugada 1 vs. Ausência de Brugada; DP: desvio padrão; IMC: índice de massa corporal; HAS: hipertensão arterial sistólica; DM: Diabetes Mellitus; IAM; infarto agudo do miocárdio prévio; DAC: doença arterial coronariana; DC: doença de Chagas; DRC doença renal crônica; DPOC: doença pulmonar obstrutiva crônica; Revasc: revascularização prévia.*

Em relação às características clínicas, os pacientes com padrão eletrocardiográfico Brugada tipo 1 ou 2 apresentaram índice de massa corporal médio significativamente menor (25,4±4,2 kg/m^2^) do que os indivíduos sem síndrome de Brugada (27,5±5,6 kg/m^2^) com p<0,001. Além disso, os pacientes com padrão eletrocardiográfico Brugada tipo 1 ou 2 apresentavam altura média maior (168,0±11,0 cm) do que aqueles sem Brugada (163,3±11,1 cm) com p=0,002. O diagnóstico de obesidade mostrou-se significativamente menos prevalente entre o grupo Brugada tipo 1 ou 2 (7,3%) em comparação com a população geral (26,4%) com p=0,001. A prevalência de obesidade também foi menor no grupo Brugada tipo 1 (9,1%) em comparação com a população geral com p=0,028.

Nenhum paciente com padrão de Brugada apresentava histórico de infarto agudo do miocárdio (IAM), doença de Chagas (DC), doença pulmonar obstrutiva crônica (DPOC) ou insuficiência renal crônica (IRC). Não houve diferenças significativas na prevalência de IAM prévio, DC, DPOC, IRC e histórico de revascularização entre os grupos estudados (Tabela 1).

## Discussão

Nosso estudo encontrou baixa prevalência do padrão eletrocardiográfico de Brugada tipo 1 entre a população da região Sul do Brasil 4,6 a cada 100.000 pacientes). Kamakura,^11^ em revisão sistemática da literatura, mostrou que a prevalência desse padrão eletrocardiográfico varia de acordo com a população e faixa etária estudada. A maior prevalência do padrão eletrocardiográfico do tipo Brugada é encontrada em alguns países asiáticos entre a população jovem, variando de 0,14 a 7,1%, com uma média estimada de 0,15%.^7^^,^^12^^–^^15^ No Japão, a prevalência varia de 4 a 122 por 10.000 habitantes.^7^^,^^12^^,^^13^^.^^16^^–^^18^ No entanto, os países ocidentais apresentam prevalência mais baixa. Estudos realizados na Europa mostram que a prevalência varia de 0 a 0,61% e estima-se uma média inferior a 0,02%.^11^^,^^19^^–^^24^ Da mesma forma, demonstrou-se que o padrão eletrocardiográfico do tipo Brugada é incomum na América do Norte. A prevalência observada em pesquisas americanas e canadenses varia de 0,012 a 0,07%.^25^^–^^27^ Em contrapartida, não temos conhecimento de estudos que demonstrem a prevalência do padrão eletrocardiográfico de Brugada na população brasileira; apenas relatos de casos e um estudo de prevalência familiar de SB.^28^^–^^31^

A prevalência encontrada neste estudo é inferior aos dados relatados na literatura e pode estar relacionada à baixa prevalência de padrão eletrocardiográfico de Brugada na população brasileira. Por ser a SB um quadro genético, é possível que as variações genéticas encontradas na população estudada possam ter influenciado a prevalência desse padrão eletrocardiográfico. Corroborando essa hipótese, Bezzina et al.^32^ demonstraram que polimorfismos genéticos relacionados à etnia podem modular a atividade da doença primária, causando mutações ou influenciando a suscetibilidade à arritmia. No mesmo estudo, uma variante genética haploide composta por seis polimorfismos relacionados ao gene SCN5A foi identificada apenas em indivíduos asiáticos, não tendo sido encontrada em indivíduos brancos e negros.^32^ No entanto, são necessários mais estudos clínicos para que se possa elucidar essa questão.

Além disso, é importante reconhecer que a grande variação da prevalência relatada na literatura pode ser devido à não padronização das definições do padrão eletrocardiográfico de Brugada utilizado antes da publicação do Relatório de Consenso sobre a Síndrome de Brugada.^1^^,^^10^ Assim, é possível que estudos realizados antes daquele ano tenham superestimado a prevalência desse padrão eletrocardiográfico. Da mesma forma, outros fatores podem ter influenciado essa variação. Por exemplo, ao contrário do Brasil, grande parte da população japonesa tem acesso a exames de saúde anuais, e diversos estudos sobre a prevalência do padrão eletrocardiográfico de Brugada foram publicados.^7^^,^^12^^,^^13^^–^^18^ Além disso, como mencionado anteriormente, a SB parece ter maior prevalência no Sudeste e Leste da Ásia. Portanto, esses dados não podem ser extrapolados para a população de países ocidentais.

A distribuição por sexo encontrada na população deste estudo mostrou predomínio do sexo masculino (81,8% em Brugada tipo 1). Nossos achados estão de acordo com os dados epidemiológicos relatados anteriormente.^11^ Estudos japoneses demonstraram que o padrão eletrocardiográfico de Brugada tem predomínio do sexo masculino, compreendendo cerca de 90% de todos os pacientes com esse padrão eletrocardiográfico. Matsuo et al.,^7^ em estudo de coorte, observaram que o percentual de homens com padrão eletrocardiográfico de Brugada foi de 84% em uma população com 43% de participantes do sexo masculino. Da mesma forma, Tsuji et al.,^18^ em uma pesquisa incluindo 26% de participantes do sexo masculino, verificaram que 84% dos ECG com padrão de Brugada foram observados em pacientes do sexo masculino. Sukabe et al.,^16^ encontraram uma prevalência de 97% de exames eletrocardiográficos com padrão de Brugada entre homens em um estudo que incluiu 79% de pacientes do sexo masculino. Esses dados sugerem que a prevalência é maior no sexo masculino e diversos estudos multicêntricos conduzidos em países ocidentais mostraram resultados semelhantes aos nossos. Isso indica que a frequência de homens com padrão eletrocardiográfico de Brugada nos países ocidentais é significativamente menor do que a população japonesa (72–80% vs. 94–96%),^33^^–^^39^

Em diversos estudos, investigou-se a maior frequência de padrão eletrocardiográfico de Brugada entre indivíduos do sexo masculino como encontrada neste trabalho, semelhante à literatura. Embora a transmissão genética ocorra na mesma proporção entre homens e mulheres, o padrão eletrocardiográfico de Brugada e as manifestações clínicas da síndrome de Brugada são observadas cerca de 8 a 10 vezes mais em homens.^40^^–^^42^ Di Diego et al.,^40^ em estudo experimental, sugeriram uma base celular para esse predomínio por meio de uma técnica de perfusão arterial em um preparo ventricular direito canino. Demonstraram que a corrente de saída transitória (I_to_), importante para a fase inicial do potencial de ação, era maior no epicárdio ventricular direito de cães machos, correspondendo assim ao mecanismo responsável pela predominância masculina do fenótipo de Brugada. Além disso, Shimizo et al.,^41^ em um estudo de caso-controle, demonstrou níveis significativamente mais elevados de testosterona em homens com padrão eletrocardiográfico de Brugada do que nos controles. Este hormônio é conhecido por aumentar as correntes de saída (I_to_). Consequentemente, espera-se a acentuação do fenótipo de Brugada como elevação do segmento ST e subsequentes episódios de FV em pacientes com síndrome de Brugada.^43^ Matsuo et al.,^44^ relataram 2 casos de pacientes assintomáticos com padrão eletrocardiográfico de Brugada persistente, nos quais o padrão eletrocardiográfico desapareceu após orquiectomia como tratamento para câncer de próstata. De forma semelhante, Yamakawa et al.,^45^ investigaram 20.387 crianças japonesas e descobriram que a prevalência do padrão eletrocardiográfico de Brugada é significativamente menor do que na população adulta. O mesmo estudo, ao comparar os sexos, encontrou uma predominância do sexo masculino que aumenta com a puberdade. Em contrapartida, Oe et al.,^46^ estudaram crianças de 6 e 7 anos e não encontraram diferença na prevalência de gênero. Esses dados sugerem que as diferenças de gênero entre os pacientes com padrão eletrocardiográfico de Brugada ocorrem após a adolescência. Este é o período em que os níveis de testosterona também aumentam.

Quanto às características clínicas deste estudo, os pacientes com padrão eletrocardiográfico de Brugada tipo 1 ou 2 tinham maior estatura, menor IMC e, consequentemente, menos diagnósticos de obesidade em relação à população geral. Quando se analisa apenas o padrão de Brugada tipo 1, observa-se uma tendência não significativa de maior estatura e menor IMC, mas a obesidade também se mostra menos prevalente do que a população em geral. Do mesmo modo, Matsuo et al.,^47^ em estudo epidemiológico caso-controle, encontraram IMC médio inferior em indivíduos com padrão de Brugada em comparação com controles. Shimizo et al.,^41^ tiveram resultados semelhantes: os participantes do estudo eram todos homens que apresentavam padrão eletrocardiográfico de Brugada e parâmetros de gordura visceral mais baixos (IMC, percentual de gordura corporal e peso corporal) do que os controles. Também observaram forte associação inversa entre a síndrome de Brugada e o IMC.^41^ Esses dados, comparados com o presente estudo, sugerem uma associação entre o baixo IMC e o fenótipo de Brugada. Seu estudo também demonstrou que todos os parâmetros de gordura visceral estavam inversamente correlacionados com os níveis de testosterona em pacientes com padrão de Brugada e controles.^41^ Já se sabe que os níveis de testosterona em homens obesos são menores em comparação com homens saudáveis da mesma faixa etária, e a diminuição dos níveis basais totais desse hormônio é um preditor independente de aumento da gordura visceral.^48^^,^^49^ Inversamente, se a perda de peso e a consequente diminuição da gordura visceral resultassem em aumento dos níveis de testosterona, a perda de peso poderia ser um gatilho para o fenótipo de Brugada, semelhante a um estado febril.^41^^,^^50^ No entanto, são necessários mais estudos para que essa questão seja elucidada.

Existem algumas limitações que precisam ser reconhecidas. A amostra pode não mostrar o verdadeiro perfil da população do Sul do Brasil, pois avaliou apenas os eletrocardiogramas do banco de dados de telemedicina de Santa Catarina, representados em sua maioria por avaliações ambulatoriais em um ambiente de atenção primária. Como esta é uma revisão retrospectiva de um banco de dados de ECG, desconhece-se a evolução clínica desses pacientes. Além disso, não foi possível realizar novo eletrocardiograma com as derivações precordiais V1 e V2 em posições mais altas ou realizar testes provocativos com bloqueadores de canais de sódio em caso de dúvida diagnóstica.^10^ Embora todos os eletrocardiogramas tenham sido avaliados por cardiologistas treinados, apenas aqueles com o descritor “síndrome de Brugada” foram reavaliados por um eletrofisiologista. Esse fato pode ter subestimado a prevalência do padrão eletrocardiográfico da síndrome, uma vez que a interpretação do ECG varia entre os diferentes observadores. O estudo não foi capaz de identificar outros critérios diagnósticos para a síndrome de Brugada, portanto não pode se estabelecer se esses pacientes apresentavam apenas padrão eletrocardiográfico de Brugada ou síndrome de Brugada. As características ecocardiográficas de pacientes com síndrome de Brugada podem oscilar ao longo do tempo e não ser encontradas em apenas um exame. Embora isso possa ter subestimado a prevalência de padrões eletrocardiográficos de Brugada em nosso estudo, essas oscilações representam um desafio para todos os estudos transversais, sendo necessários estudos de coorte para verificar esses dados. No entanto, o viés do estudo não invalida os resultados.

## Conclusão

Em conclusão, nosso estudo identificou baixa prevalência do padrão eletrocardiográfico da síndrome de Brugada em Santa Catarina. As características relacionadas dos pacientes com padrão eletrocardiográfico de Brugada tipo 1 ou 2 encontradas neste estudo foram: sexo masculino, maior estatura média, menores valores médios de IMC e, consequentemente, menos diagnósticos de obesidade em relação à população geral. As características relacionadas dos pacientes com apenas o padrão eletrocardiográfico de Brugada tipo 1 foram: sexo masculino e menos obesidade do que a população em geral.
