# Supplementary material for: Prevalência e Características Relacionadas de Pacientes com Eletrocardiograma com Padrão de Brugada em Santa Catarina, Brasil
Source: Arq Bras Cardiol. 2021 Aug 9;117(2):343–9. [Article in Portuguese] doi: 10.36660/abc.20190542 (PMC8395791; doi:10.36660/abc.20190542)
Supplement: Translation

# Translation: Prevalence and Related Characteristics of Patients with Brugada Pattern Electrocardiogram in Santa Catarina, Brazil

**Authors:** Mariana Sarmento Militz, Andrei de Souza Inacio, Harley Miguel Wagner, Aldo von Wangenheim, Alexander Romeno Janner Dal Forno, Daniel Medeiros Moreira

**Affiliations:** 1 PalhoçaSC Brazil Universidade do Sul de Santa Catarina, Palhoça, SC - Brazil.; 2 FlorianópolisSC Brazil Universidade Federal de Santa Catarina, Florianópolis, SC - Brazil.; 3 São JoséSC Brazil Instituto de Cardiologia de Santa Catarina, São José, SC - Brazil.; 4 FlorianópolisSC Brazil SOS Cardio, Florianópolis, SC - Brazil.

**Keywords:** Brugada Syndrome, Electrocardiography/methods, Obesity, Arrythimias, Heredity, Epidemiology

## Abstract

**Background:**

Brugada Syndrome is an inherited arrhythmogenic disorder characterized by the presence of specific electrocardiographic features with or without clinical symptoms. The patients present increased risk of sudden death due to ventricular fibrillation. The prevalence of this electrocardiographic pattern differs according to the studied region. However, epidemiological information including the Brazilian population is scarce.

**Objectives:**

To assess the prevalence of the electrocardiographic pattern of Brugada syndrome and the epidemiological profile associated with it.

**Methods:**

Cross-sectional study that included 846,533 ECG records of 716,973 patients from the electrocardiogram (ECG) database from the Santa Catarina Telemedicine Network over a 4-year period. All tests were 12-lead conventional ECG (without V1 and V2 in high positions). The tests revealing “Brugada Syndrome” diagnosis (Types 1 and 2) were reviewed by a cardiac electrophysiologist. The level of significance was set at p<0.05.

**Results:**

In total, 83 patients had a pattern potentially consistent with Brugada-type pattern ECG. Of these, 33 were confirmed having Brugada-type 1, and 22 with type 2 ECG after reevaluation. The prevalence of Brugada-type 1 ECG was 4.6 per 100,000 patients. Brugada-type 1 ECG was associated with the male gender (81.8% vs. 41.5%, p<0.001) and a lower prevalence of obesity diagnosis (9.1% vs. 26.4%, p=0.028).

**Conclusions:**

This study showed low prevalence of Brugada-type ECG in Southern Brazil. The presence of Brugada-type 1 ECG was associated with the male gender and lower prevalence of obesity diagnosis comparing to the general population.

## Introduction

Brugada syndrome (BS) is an inherited arrhythmogenic disorder characterized by the presence of specific electrocardiographic features with or without clinical symptoms. The patients are mostly young adults and present an increased risk of sudden death due to ventricular fibrillation (VF).^1^^,^^2^ This clinical entity was first described in 1992 when the Brugada brothers reported 8 cases of patients with idiopathic VF who had aborted sudden cardiac death. Those patients presented ECGs showing ST-segment elevation in the right precordial leads in the absence of structural heart disease, electrolyte disturbance, or ischemia.^3^

The BS belongs to a group of channelopathies caused by mutations occurring in genes that encode or regulate sodium channels in the cardiac muscle.^4^ This genetic transmission pattern has an autosomal dominant characteristic with mutations of SCN5A and SCN10A genes linked to the Brugada phenotype.^4^^,^^5^

The diagnosis of BS can be made using 12-lead ECG demonstrating elevation of the J-point in the right precordial leads.^1^^,^^2^ However, the true prevalence of the syndrome among the general population is complicated to estimate because some patients have a transient Brugada-type ECG.^2^ It is believed that BS is responsible for 2 to 12% of all sudden deaths, and at least 20% of deaths in patients with structurally normal hearts.^1^^,^^2^ Studies conducted in Asian countries have shown a higher prevalence of Brugada-type ECG compared to other regions.^6^^,^^7^ On the other hand, epidemiological information including the Brazilian population is scarce. This study was carried out to identify the prevalence and related characteristics of patients with Brugada-type ECG in Santa Catarina-Brazil.

## Methods

In this study, we included 846,533 ECG records of 716,973 patients from the electrocardiogram (ECG) database from the Santa Catarina Telemedicine Network. This is an electronic tool that helps healthcare professionals to view and diagnose image tests remotely. The network is connected to primary healthcare centers and remote regions from more than 250 cities in the State of Santa Catarina, Brazil. It is estimated that the system processes more than 17,000 ECGs monthly.^8^

Eligible individuals were all the patients who underwent a twelve-lead ECG exam and had their records processed in the Santa Catarina Telemedicine Network from August 2010 to December 2015.

Most tests were performed on an outpatient basis, especially in primary healthcare units with indication for cardiovascular assessment in primary care (data not shown). All tests from our database were evaluated by a trained cardiologist and all those identified with the descriptor “Brugada syndrome” as a diagnosis were reviewed by a second cardiologist specializing in electrophysiology.

We used the Consensus Report of the *Current electrocardiographic criteria for diagnosis of Brugada pattern* as a basis for identifying the electrocardiographic pattern.^9^ The ECGs were reevaluated using the following criteria: Type 1 (coved pattern) with initial ST-elevation ≥2 mm slowly descending and concave or rectilinear with respect to the isoelectric baseline, with negative symmetric T wave in V1-V2, and Type 2 (saddle back pattern) — high take-off (r') ≥2 mm with respect to the isoelectric line and followed by ST-elevation convex with respect to the isoelectric baseline with ≥0.05 mV elevation with positive/flat T wave in V2 and T wave variable in V1.

Only the first confirmed test of each patient was considered. For comparison, we used the first test of all the other patients who underwent ECG in the same period and were not diagnosed with BS. As this is a retrospective study, without direct access to clinical data of patients, it aimed to estimate the prevalence of the electrocardiographic pattern of Brugada syndrome. However, it is not possible to determine the true prevalence of the syndrome, since those tests that were phenocopies of the Brugada syndrome, for instance,^10^ cannot be ruled out. We also compared epidemiological data between the groups of tests such as age, sex and prevalence of previous diseases, including diabetes, dyslipidemia, hypertension, chronic kidney disease, coronary artery disease, Chagas disease and previous acute myocardial infarction.

### Statistical Analysis

Statistical analysis was performed using the SPSS 13.0 software for Windows (SPSS Inc., Chicago, IL, USA). We used the Kolmogorov-Smirnov test to assess the normality of continuous variables: all of them had normal distribution and, then, were presented as mean and standard deviation. Categorical variables were presented by absolute numbers and percentages. The quantitative variables between the study groups were evaluated using the unpaired Student's *t* test. Fisher's exact test was used to test the association between proportions. The level of significance was set at p<0.05.

## Results

This study included 846,533 tests from 716,973 patients. Among them, 83 patients had a pattern potentially consistent with Brugada-type ECG. We excluded 129,560 tests because they belonged to the same patients. After reassessment of the tests with BS diagnoses by an expert in electrophysiology, it was possible to confirm 55 ECGs with Brugada pattern. Of these, 33 tests were diagnosed as Brugada type 1 pattern and 22 tests as Brugada type 2 pattern. The comparison group (with no diagnosis of BS) had 716,918 patients.

Prevalence of Brugada type 1 or 2 ECG was 7.6 per 100,000 patients. Prevalence of Brugada type 1 ECG pattern and Brugada type 2 pattern were 4.6 and 3.0 per 100,000 patients, respectively.

The sample characteristics are presented in Table 1. The mean age of patients in the Brugada type 1 or 2 group was 48.0±16.0 years. There were 78.2% of males in the Brugada type 1 or 2 group and 81.1% in the Brugada type 1 group, showing a significantly higher proportion than the general population evaluated (41.5%) with p<0.001 for comparison.

**Table 1:** Demographic and clinical variables of the study population

Variables		Brugada pattern types 1 and 2 n (%) mean ± SD	Brugada pattern type 1 n (%) mean ± SD	No Brugada pattern n (%) mean ± SD	p value*	p value†
Gender						
	Male	43 (78,2)	27 (81,8)	297131 (41,5)	<0,001	<0,001
	Female	12 (21,8)	6 (18,2)	419603 (58,5)	-	-
Age (years)		48.0±16,0	48.0±15,5	50.0±19,6	0,431	0,568
Height (cm)		168.0±11,0	166.8±12,6	163.3±11,1	0,002	0,067
Weight (kg)		72.6±15,6	72.7±17,2	73.6±17,4	0,664	0,776
BMI (kg/cm2)		25.4±4,2	25.7±4,5	27.5±5,6	0,001	0,084
HBP		19 (34,5)	12 (36,4)	253469 (35,4)	0,994	1,000
Obesity		4 (7,3)	3 (9,1)	188961 (26,4)	0,001	0,028
DM		6 (10,9)	3 (9,1)	54732 (7,6)	0,312	0,738
Smoking		1 (1,8)	1 (3,0)	51645 (7,2)	0,185	0,730
AMI		0 (0,0)	0 (0,0)	6960 (1,0)	>0,999	>0,999
Dyslipidemia		2 (3,6)	1 (3,0)	63229 (8,8)	0,234	0,361
CAD		6 (11,0)	3 (9,1)	92999 (13,0)	0,841	0,794
CD		0 (0,0)	0 (0,0)	359 (0,1)	>0,999	>0,999
CKD		0 (0,0)	0 (0,0)	2819 (4,0)	>0,999	>0,999
COPD		0 (0,0)	0 (0,0)	8837 (1,2)	>0,999	>0,999
Revasc		1 (1,8)	1 (3,0)	4962 (6,0)	0,268	0,171

* *Brugada types 1 and 2 vs. No Brugada,*

† *Brugada 1 vs. No Brugada; SD: standard deviation; BMI: body mass index; HBP: high blood pressure; DM: Diabetes Mellitus; AMI: previous acute myocardial infarction; CAD: coronary artery disease; CD: Chagas disease; CKD: chronic kidney disease; COPD: chronic obstructive pulmonary disease; Revasc: previous revascularization*.

Regarding the clinical characteristics, patients with Brugada type 1 or 2 ECG had a significantly lower mean body mass index (25.4±4.2 kg/m^2^) than individuals without Brugada (27.5 ± 5.6 kg/m^2^) with p <0.001. In addition, patients with Brugada type 1 or 2 ECG showed a greater mean height (168.0±11.0 cm) than those without Brugada (163.3±11.1 cm) with p=0.002. Diagnosis of obesity was significantly less prevalent among the Brugada type 1 or 2 group (7.3%) compared to general population (26.4%) with p=0.001. The prevalence of obesity was also lower in the Brugada type 1 group (9.1%) compared to the general population with p=0.028.

No patient with Brugada pattern had previous acute myocardial infarction (AMI), Chagas disease (CD), chronic obstructive pulmonary disease (COPD) or chronic renal failure (CRF). There were no significant differences in the prevalence of previous AMI, CD, COPD, CRF and history of revascularization among the study groups (Table 1).

## Discussion

Our study found a low prevalence of Brugada type 1 ECG pattern among southern Brazilians (4.6 per 100,000 patients). Kamakura,^11^ in a systematic literature review, showed that the prevalence of this ECG pattern varies according to the population and age group studied. The highest prevalence of Brugada type ECG is found in some Asian countries amongst young adults, ranging from 0.14 to 7.1%, with an estimated average of 0.15%.^7^^,^^12^^–^^15^ In Japan, the prevalence ranges from 4 to 122 per 10,000 inhabitants.^7^^,^^12^^,^^13^^,^^16^^–^^18^ However, Western countries have a lower prevalence. Studies conducted in Europe have shown that the prevalence varies from 0 to 0.61% and an average of less than 0.02% is estimated.^11^^,^^19^^–^^24^ Likewise, the Brugada-type ECG has been shown to be uncommon in North America. The prevalence observed in American and Canadian surveys ranges from 0.012 to 0.07%.^25^^–^^27^ In contrast, we are not aware of studies that demonstrate the prevalence of Brugada-type ECG in the Brazilian population, only case reports and a family prevalence study of BS.^28^^–^^31^

The prevalence found in this study is lower than data reported in the literature and may be related to a low prevalence of Brugada-type ECG among the Brazilian population. Since the BS is a genetic condition, it is possible that the genetic variations found in the study population may have influenced the prevalence of this electrocardiographic pattern. Corroborating this hypothesis, Bezzina et al.^32^ demonstrated that ethnicity-related genetic polymorphisms can modulate primary disease activity causing mutations or influencing susceptibility to arrhythmia. In the same study, a haploid genetic variant consisting of six SCN5A gene-related polymorphisms was identified only in Asian subjects and was not present in white and black individuals.^32^ However, more studies are needed to clarify this issue.

In addition, it is important to recognize that the wide variation of the prevalence reported in the literature may be due to the non-standardization of Brugada pattern ECG definitions used before the publication of the Brugada Syndrome Consensus Report.^1^^,^^10^ Thus, it is possible that studies conducted before that year may have overestimated the prevalence of this electrocardiographic pattern. Likewise, other factors may have influenced this variation. For example, unlike Brazil, a large part of the Japanese population has access to annual health examinations, and several studies about the prevalence of Brugada-type ECG have been published.^7^^,^^12^^,^^13^^,^^18^ Moreover, as previously mentioned, the BS appears to have a higher prevalence in Southeast and East Asia, so this data cannot be extrapolated to the population of Western countries.

The gender distribution found in the population of this study showed a predominance of males (81.8% in Brugada type 1). Our findings are in agreement with epidemiological data previously reported.^11^ Japanese studies have shown that Brugada-type ECG has a predominance of men, comprising about 90% of all patients with this electrocardiographic pattern. Matsuo et al.,^7^ in a cohort study, observed that the percentage of men with Brugada-type ECG was 84% in a population of 43% of male participants. Similarly, Tsuji et al.,^18^ in a survey including 26% of male participants, found that 84% of Brugada-type ECG were observed in male patients. Sukabe et al.^16^ found a 97% prevalence of Brugada-type ECG among men in a study comprising of 79% male patients*.* These data suggest that the prevalence is higher among males and many multicenter studies conducted in Western countries have shown similar results to ours. This indicates that the frequency of men with Brugada-type ECG in Western countries is significantly lower than the Japanese population (72–80% vs. 94–96%).^33^^–^^39^

The higher frequency of Brugada-type ECG among male individuals found in this paper, similar to the literature, has been investigated in several studies. Although genetic transmission occurs in the same proportion between men and women, the Brugada-type ECG and the clinical manifestations of the Brugada syndrome are observed around 8 to 10 times more in men.^40^^–^^42^ Di Diego et al.,^40^ in an experimental study, suggested a cellular basis for this predominance using an arterial perfusion technique in a canine right ventricle preparation. They demonstrated that the transient outward current (I_to_), which is important to the initial phase of the action potential, was higher in the right ventricular epicardium of male dogs, thus corresponding to the mechanism responsible for the male predominance of the Brugada phenotype. In addition, Shimizo et al.,^41^ in a case-control study, demonstrated significantly higher testosterone levels in men with Brugada-type ECG than controls. This hormone is known to increase outward currents (I_to_). Consequently, accentuation of the Brugada phenotype such as ST-segment elevation and subsequent VF episodes in patients with Brugada syndrome,^43^ is expected. Matsuo et al.^44^ reported 2 cases of asymptomatic patients with persistent Brugada-type ECG in which the electrocardiographic pattern disappeared after orchiectomy as a treatment for prostate cancer. Similarly, Yamakawa et al.^45^ investigated 20,387 Japanese children and found that the prevalence of Brugada ECG is significantly lower than in the adult population. The same study, in a comparison of genders, found a male predominance that increases with puberty. In contrast, Oe et al.^46^ studied 6 and 7-year-old children and found no difference in gender prevalence. These data suggest that the gender differences among patients with Brugada-type ECG occurs after adolescence. This is the period when testosterone levels also increase.

As for the clinical characteristics of this study, patients with Brugada-type 1 or 2 ECG were taller, had lower BMI and, as a consequence, less diagnosis of obesity compared to the general population. When only the Brugada type 1 pattern is analyzed, there is a non-significant trend towards higher height and lower BMI, but obesity has also been shown to be less prevalent than the general population. Likewise, Matsuo et al.,^47^ in an epidemiological case control study, found lower mean BMI in individuals with Brugada pattern than controls. Shimizo et al.^41^ had similar results: the study participants were all men who presented Brugada-type ECG and lower visceral fat parameters (BMI, body fat percentage and body weight) than controls. They also observed a strong inverse association between Brugada syndrome and BMI.^41^ These data, compared to the present study, suggest an association between low BMI and Brugada phenotype. Their study also demonstrated that all the visceral fat parameters were inversely correlated with testosterone levels in both patients with Brugada pattern and controls.^41^ It is already known that testosterone levels in obese men are lower compared to healthy men of the same age group, and the decrease in total baseline levels of this hormone is an independent predictor of increased visceral fat.^48^^,^^49^ On the contrary, if weight loss and consequent decrease in visceral fat would result in an increase in testosterone levels, weight loss could be a trigger for the Brugada phenotype, similar to a febrile state.^41^^,^^50^ However, this question needs further studies to be elucidated.

There are some limitations that need to be acknowledged. The sample may not show the true profile of the southern Brazilian population, since it only evaluated the electrocardiograms from the Santa Catarina Telemedicine database, represented mostly by outpatient evaluations in a primary care setting. Since this is a retrospective review of an ECG database, the clinical outcome of these patients is unknown. In addition, it was not possible to perform a new electrocardiogram with precordial leads V1 and V2 in higher positions or to perform provocative tests with sodium channel blockers in cases of diagnostic doubt.^10^ Although all electrocardiograms were evaluated by trained cardiologists, only those with the descriptor “Brugada syndrome” were reevaluated by an electrophysiologist: this fact may have underestimated the prevalence of the electrocardiographic pattern of the syndrome, since ECG interpretation varies among different observers. The study was unable to identify other diagnostic criteria for Brugada syndrome, so it cannot be established whether these patients only had Brugada-type ECG or Brugada syndrome. The ECG features of patients with Brugada syndrome may fluctuate over time and not be found in only one examination. Although this may have underestimated the prevalence of Brugada-type ECG in our study, these fluctuations represent a challenge for all cross-sectional studies, and cohort studies are required to verify these data. However, the study bias does not invalidate the findings.

## Conclusion

In conclusion, our study identified a low prevalence of the electrocardiographic pattern of Brugada syndrome in Santa Catarina. The related characteristics of patients with Brugada-type 1 or 2 ECG found in this study were: male gender, greater mean height, lower mean BMI values and, as a consequence, less diagnosis of obesity compared to the general population. The related characteristics of patients with only Brugada-type 1 ECG were: male gender and less obesity than the general population.
